# Supplementary material for: Large-scale sequencing of SARS-CoV-2 genomes from one region allows detailed epidemiology and enables local outbreak management
Source: Microb Genom. 2021 Jun 29;7(6):000589. doi: 10.1099/mgen.0.000589 (PMC8461472; doi:10.1099/mgen.0.000589)
Supplement: Supplementary material 1 [file mgen-7-0589-s001.pdf]

Supplementary Figures for the manuscript “Large scale sequencing of SARS-CoV-2 genomes from one region allows detailed epidemiology and enables local outbreak management”

Supplementary Figures

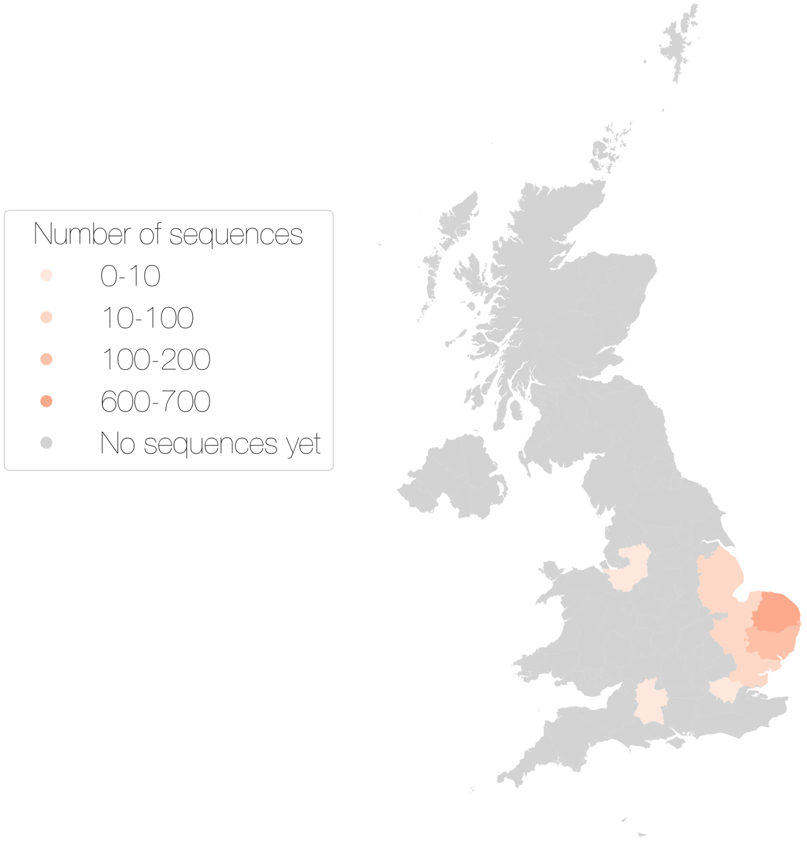

Supplementary Figure 1: The reported home address of the cases sampled, anonymised to region level.

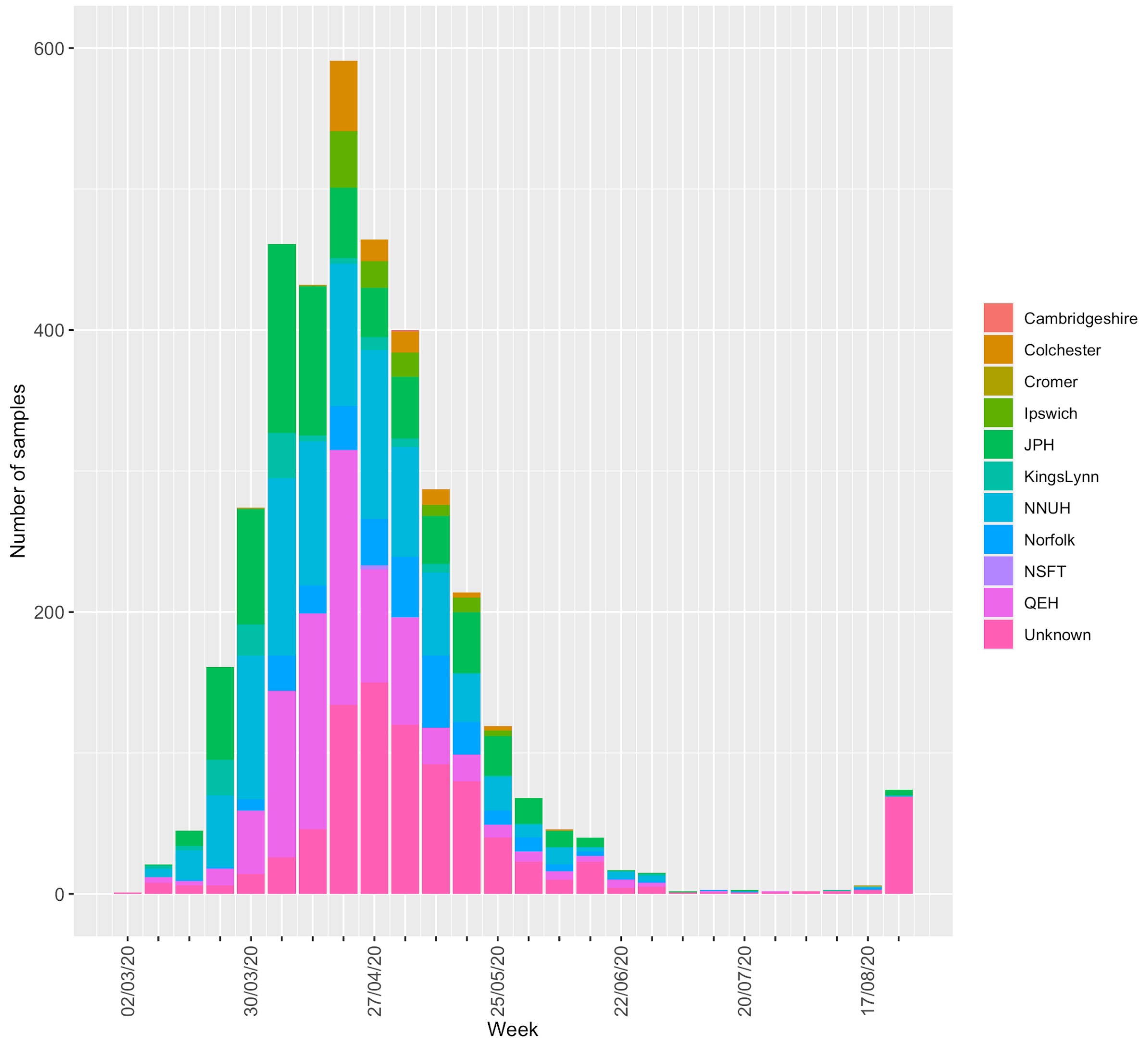

Supplementary Figure 2: Total number of positive samples per week, broken down by the locality from which they were collected. If specific hospital information was not available, geographical location at the finest resolution was used. Not all samples were sequenced and a single individual may have been sampled multiple times.

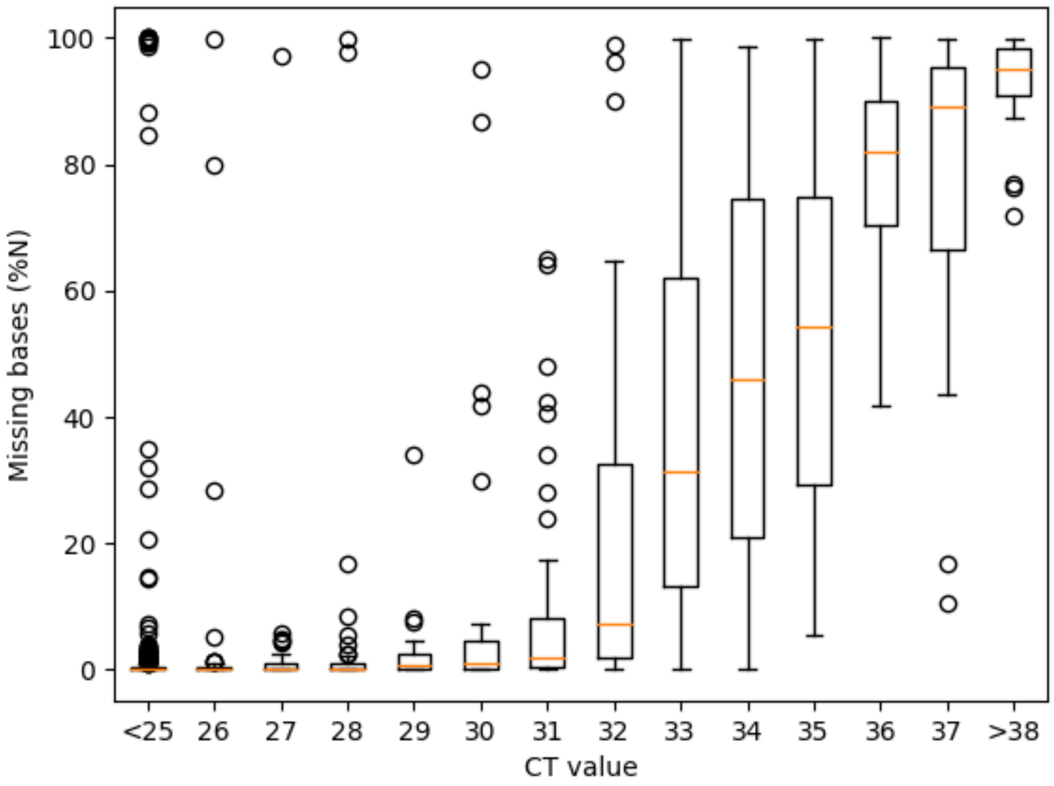

Supplementary Figure 3: The percentage of missing bases in each consensus genome against the Ct value, where 0 means the consensus genome has no missing bases compared to the Wuhan Hu-1 reference genome (accession MN908947.3) and 100 means the consensus genome is missing every base.

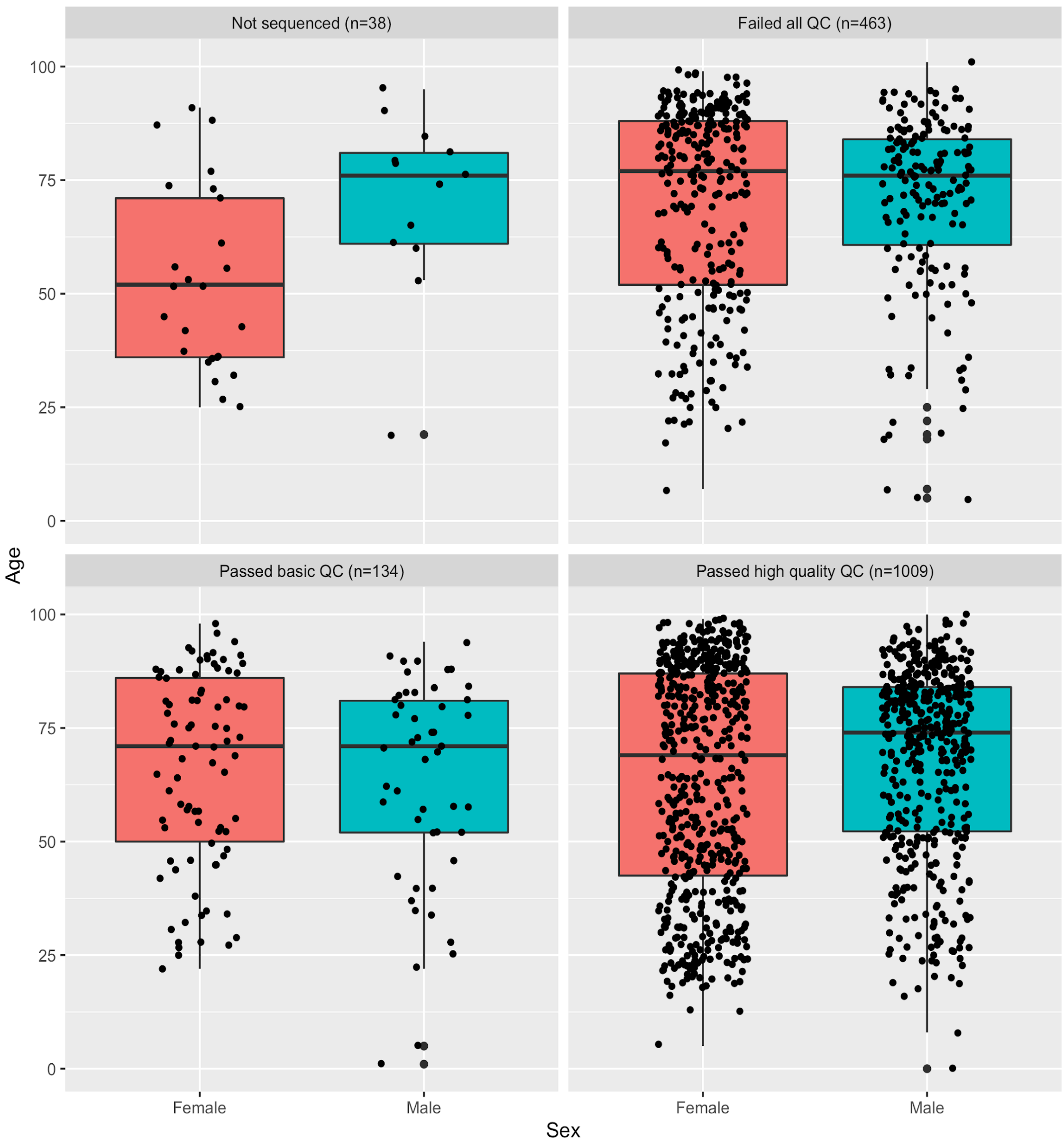

Supplementary Figure 4: The samples, for which age and sex information were available, categorised by age, sex of the SARS-CoV-2 positive individual and viral genome sequencing quality. 38 samples had metadata but the physical sample was not available for sequencing.

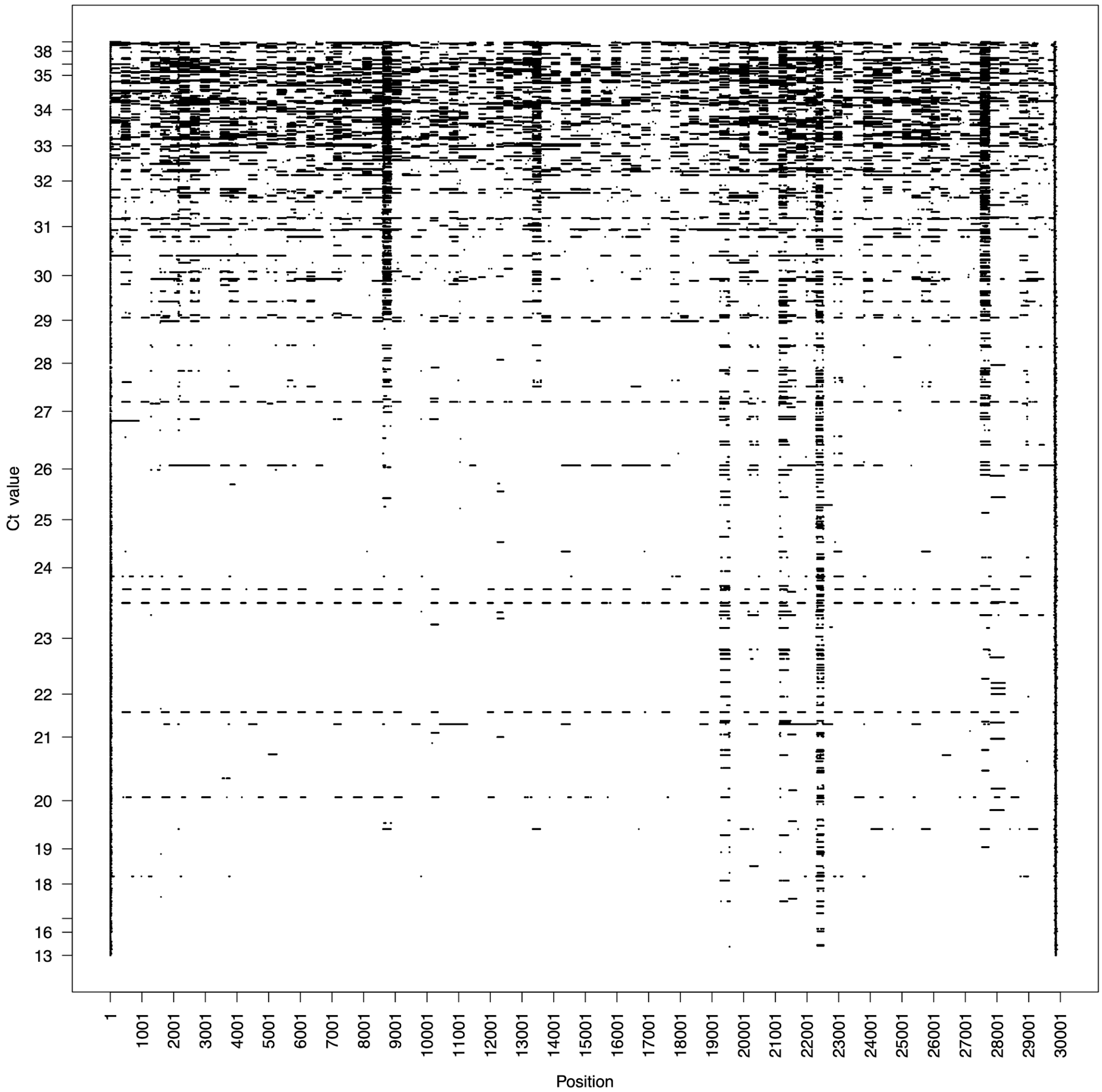

Supplementary Figure 5: The consensus genomes samples against the Wuhan Hu-1 reference genome (accession MN908947.3), where a black line indicates the absence of data (N) in a consensus genome and white indicates the presence of data.

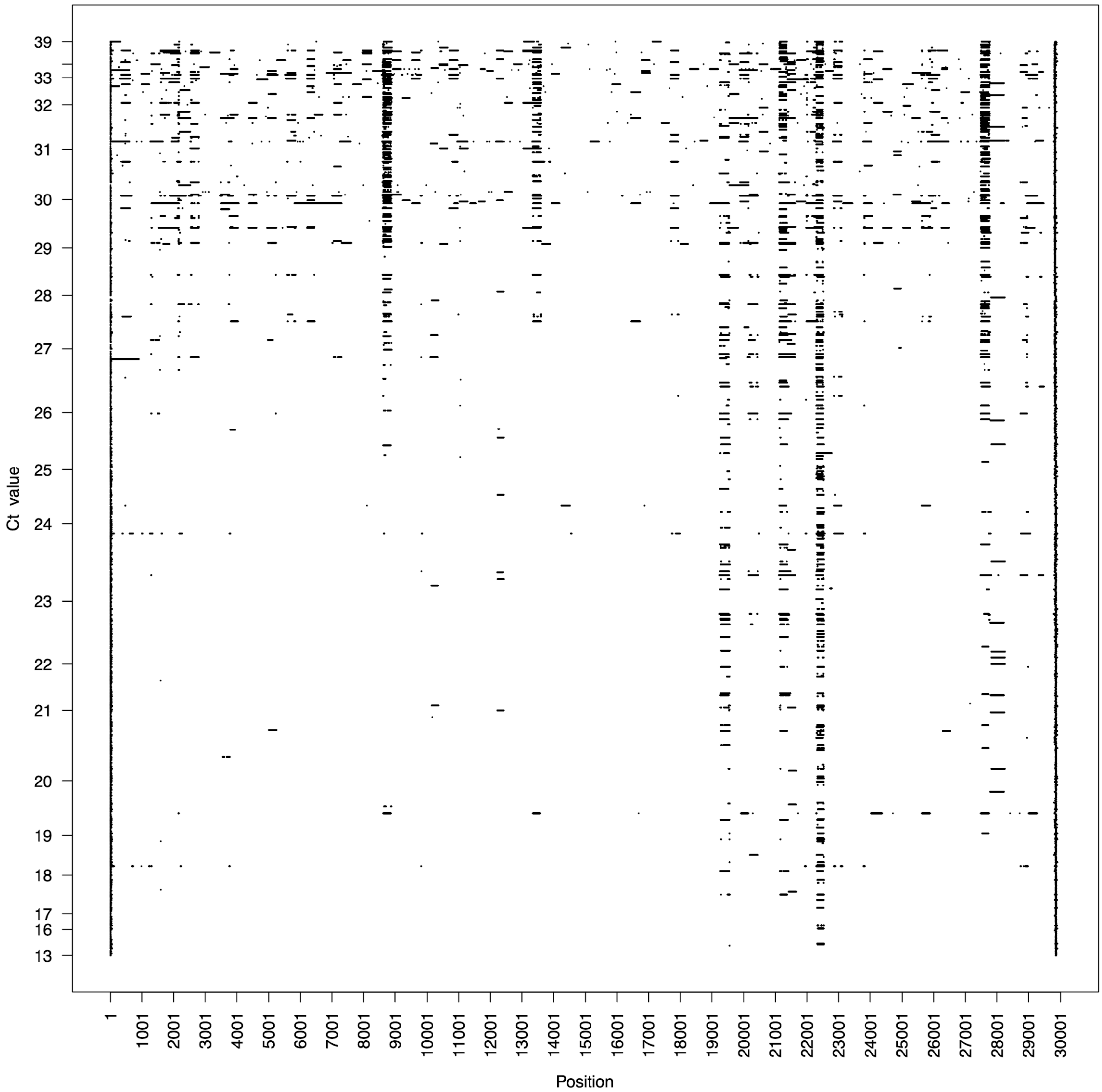

Supplementary Figure 6: The consensus genomes of all samples passing GISIAD QC against the Wuhan Hu-1 reference genome, where a black line indicates the absence of data (N) in a consensus genome.

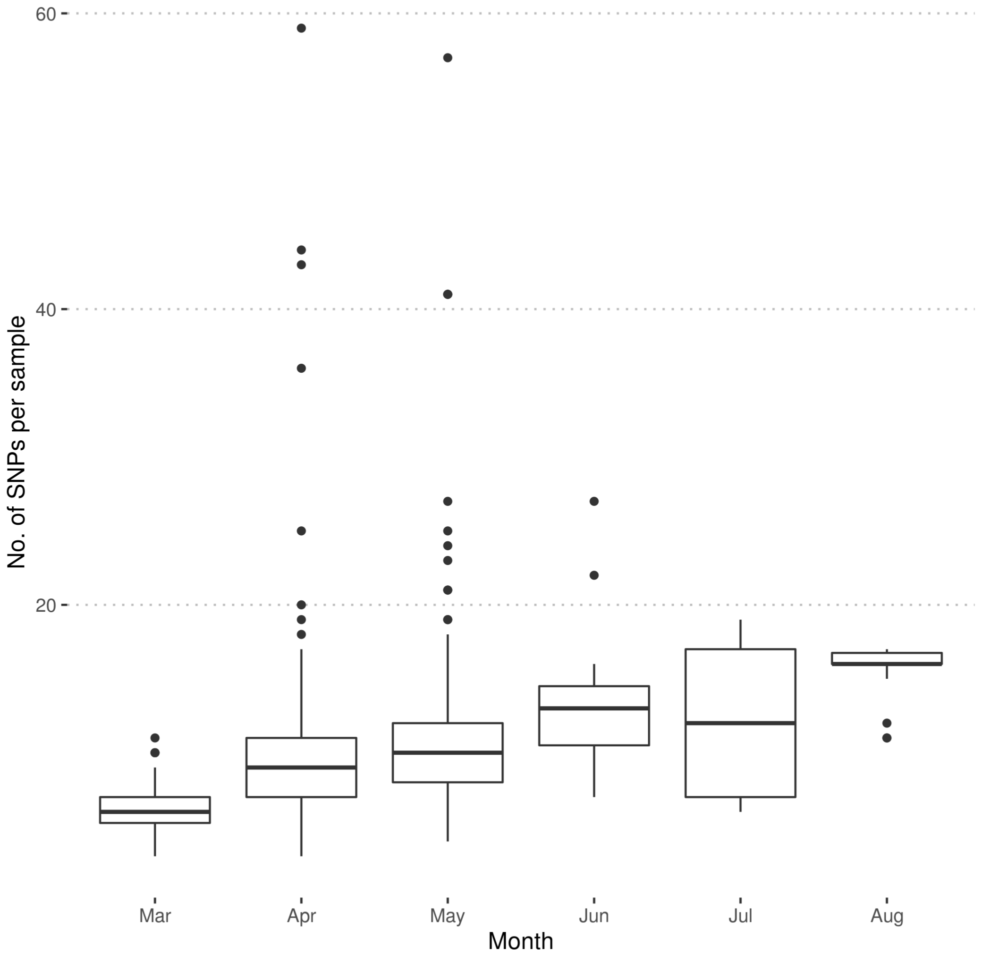

Supplementary Figure 7: The number of SNPs per sample per month of collection compared to the Wuhan Hu-1 reference genome.

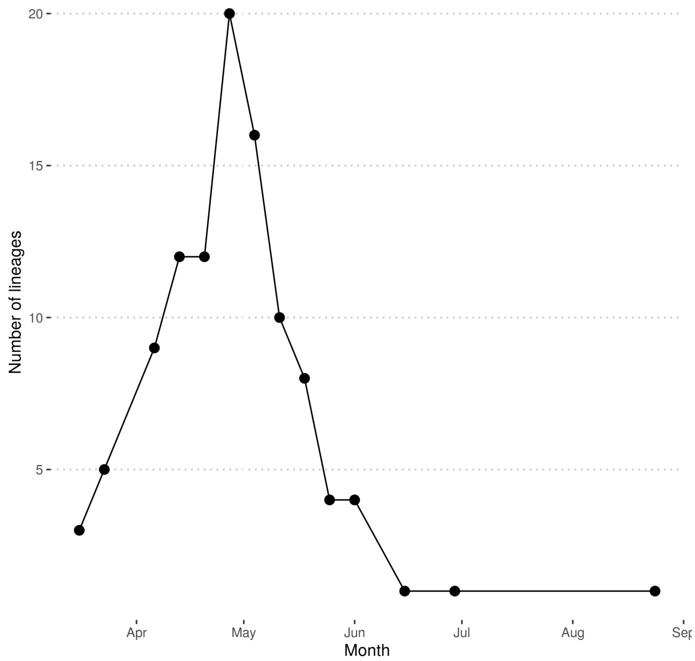

Supplementary Figure 8: The number of UK lineages observed in a given week in Norfolk.

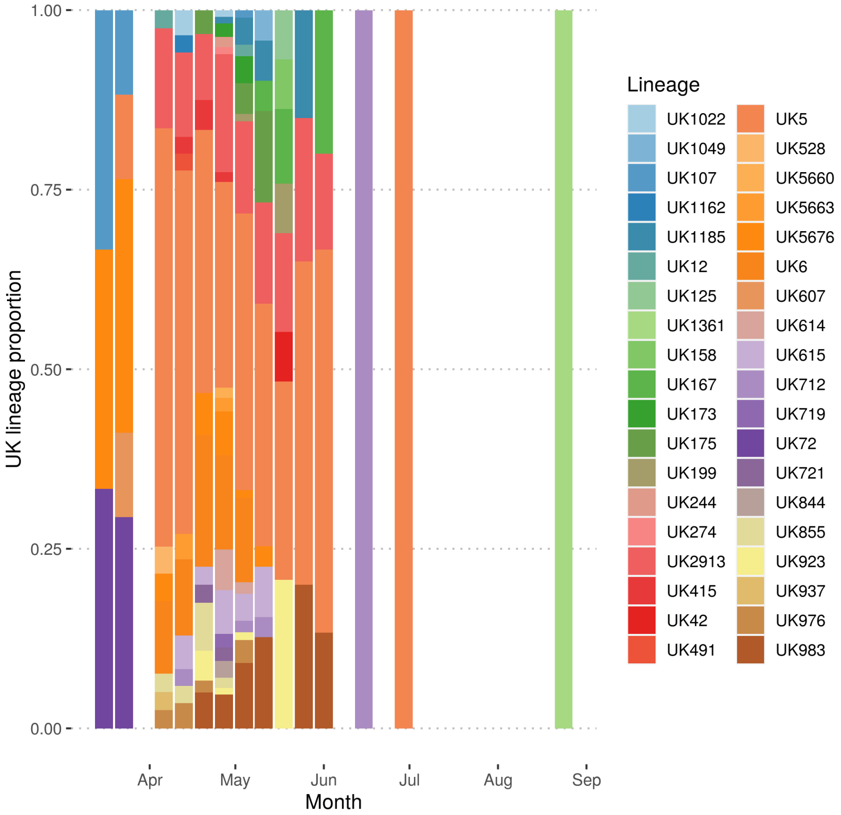

Supplementary Figure 9: The proportion of samples for each UK lineage identified in Norfolk where the lineage contained two or more representatives.

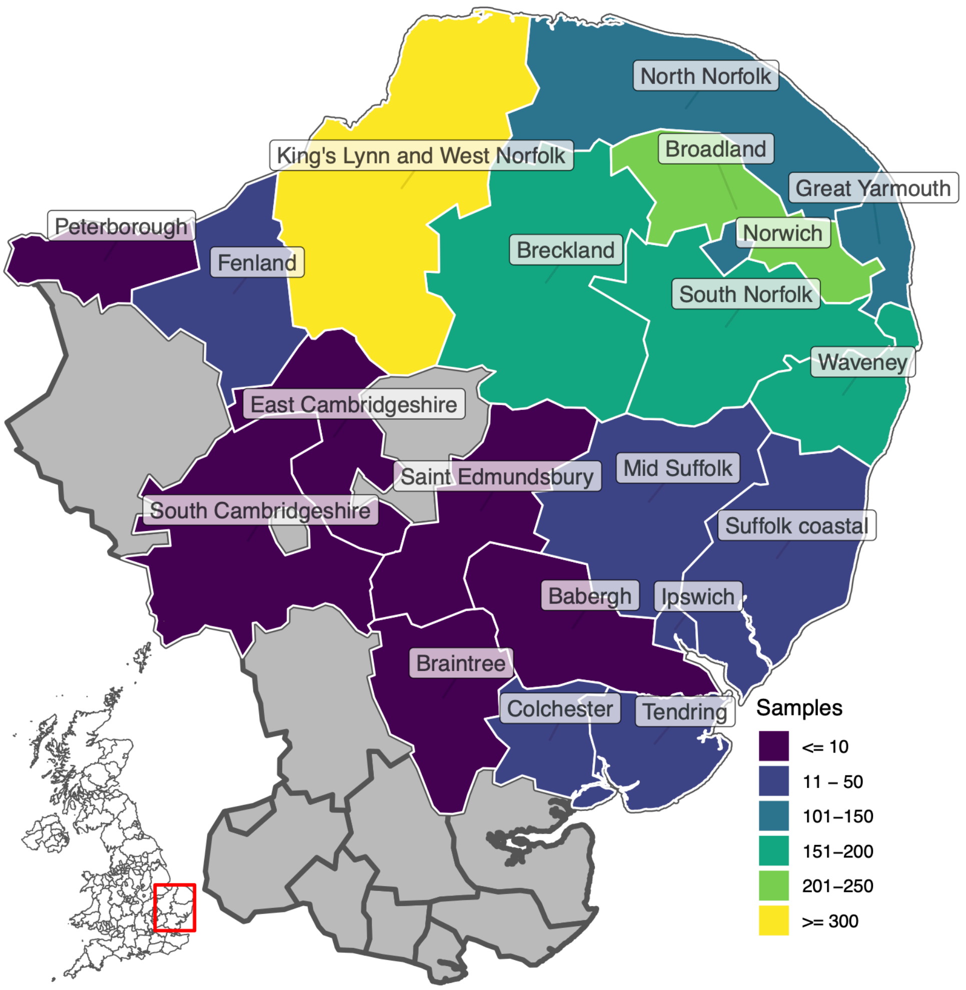

Supplementary Figure 10: Number of cases sequenced per locality in the East of England.
